# Elevated Systemic IL-10 Levels Indicate Immunodepression Leading to Nosocomial Infections after Aneurysmal Subarachnoid Hemorrhage (SAH) in Patients

**DOI:** 10.3390/ijms21051569

**Published:** 2020-02-25

**Authors:** Shafqat Rasul Chaudhry, Ulf Dietrich Kahlert, Thomas Mehari Kinfe, Alf Lamprecht, Mika Niemelä, Daniel Hänggi, Sajjad Muhammad

**Affiliations:** 1 Department of Neurosurgery, University Hospital Bonn, University of Bonn, Sigmund-Freud Str. 25, D-53105 Bonn, Germany; shafqat.scps@stmu.edu.pk or; 2 Department of Pharmaceutics, Institute of Pharmacy, University of Bonn, Gerhard-Domagk-Strasse 3, D-53121 Bonn, Germany; 3 College of Pharmaceutical Sciences, Shifa-Tameer-e-Millat University, Pitras Bukhari Road H-8/4, Islamabad 44000, Pakistan; 4 Department of Neurosurgery, Medical Faculty, Heinrich-Heine-University, Moorenstrasse 5, 40225 Düsseldorf, Germany; ulf.kahlert@med.uni-duesseldorf.de (U.D.K.); daniel.haenggi@med.uni-duesseldorf.de (D.H.); 5 Division of Functional Neurosurgery and Stereotaxy, Friedrich-Alexander University (FAU) Erlangen-Nürnberg, 91054 Erlangen, Germany; thomasmehari.kinfe@uk-erlangen.de; 6 Department of Neurosurgery, University of Helsinki and Helsinki University Hospital, 00029 Helsinki, Finland; mika.niemela@hus.fi

**Keywords:** subarachnoid hemorrhage, cytokine, anti-inflammatory, early brain injury, stroke, aneurysm, inflammation, cerebral vasospasm, clinical outcome, complications

## Abstract

Background: Aneurysmal subarachnoid hemorrhage (SAH) is a highly complex disease with very high mortality and morbidity. About one-third of SAH patients suffer from systemic infections, predominantly pneumonia, that can contribute to excess mortality after SAH. Immunodepression is probably the most important mechanism leading to infections. Interleukin-10 (IL-10) is a master regulator of immunodepression, but it is still not clear if systemic IL-10 levels contribute to immunodepression, occurrence of infections and clinical outcome after SAH. Methods: This explorative study included 76 patients with SAH admitted to our neurointensive care unit within 24 h after ictus. A group of 24 patients without any known intracranial pathology were included as controls. Peripheral venous blood was withdrawn on day 1 and day 7 after SAH. Serum was isolated by centrifugation and stored at −80 °C until analysis. Serum IL-10 levels were determined by enzyme-linked immunoassay (ELISA). Patient characteristics, post-SAH complications and clinical outcome at discharge were retrieved from patients’ record files. Results: Serum IL-10 levels were significantly higher on day 1 and day 7 in SAH patients compared to controls. Serum IL-10 levels were significantly higher on day 7 in patients who developed any kind of infection, cerebral vasospasm (CVS) or chronic hydrocephalus. Serum IL-10 levels were significantly higher in SAH patients discharged with poor clinical outcome (modified Rankin Scale (mRS) 3–6 or Glasgow Outcome Scale (GOS) 1–3). Conclusion: Serum IL-10 might be an additional useful parameter along with other biomarkers to predict post-SAH infections.

## 1. Introduction

Aneurysmal subarachnoid hemorrhage (SAH) is associated with a mortality rate of around 40–50% [1,2]. Despite successful aneurysm obliteration from the systemic circulation, a very high proportion of SAH patients confront life-threatening complications, and more than one-third of SAH patients develop systemic or local infections [3,4,5,6,7,8,9]. Post-SAH immunodepression may influence occurrence of infections. Interleukin (IL)-10 has been shown to be the master regulator of immunity, infection and immunodepression [10,11]. IL-10 is secreted by almost all types of immune cells under different conditions and is co-induced with proinflammatory cytokines via pathways that have negative regulatory feedback loops, in order to limit damage to the host [12]. For example, IL-10 inhibits the production of proinflammatory cytokines such as IL-1α, IL-1β, IL-6, IL-12, IL-18, G-CSF, TNF-α, PAF and LIF, as well as chemokines such as MCP-1, MCP-5, MIP-1α, MIP-1β, RANTES, CXCL8, IP-10, MIP-2 and KC [12,13]. Interestingly, IL-10 promotes differentiation of IL-10-secreting regulatory T (Treg cells) cells with immunosuppressive properties [14]. Human IL-10, a homodimer of 35kDa, is encoded on chromosome 1 and signals by binding to the IL-10 receptor (IL-10R) through a downstream pathway dependent upon signal transducer and activator of transcription 3 (STAT3) [12,15].

In different central nervous system (CNS) pathologies, including multiple sclerosis, stroke, and Alzheimer’s disease, IL-10 has been shown to be upregulated. This upregulation limits inflammation via reduction of proinflammatory cytokine synthesis, decreasing the expression of cytokine receptors and also inhibiting receptor activation by promoting neuronal and glial cell survival [16]. In SAH patients, IL-10 levels in cerebrospinal fluid (CSF) and extracellular fluid from microdialysis remain unchanged [17,18,19]. Systemic levels of IL-10 reflected a similar pattern in other CNS pathologies [17].

In SAH, IL-10 levels were significantly elevated after SAH and were associated with general cerebral edema [20], which is a prognostic factor for poor clinical outcome in patients. However, detailed preclinical and clinical studies investigating the association between serum IL-10 levels and post-SAH infections are still lacking [21]. The current study was planned to investigate contribution of systemic IL-10 levels during early and delayed brain injury towards the development of post-SAH infections and clinical outcome.

## 2. Results

### 2.1. Aneurysmal SAH Led to Elevated Systemic IL-10 Levels

**Figure 1:**
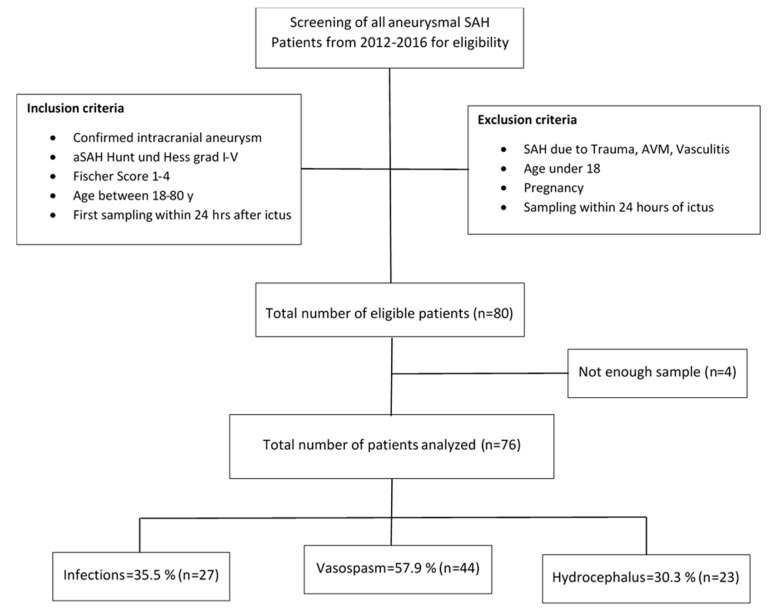
Flow diagram representing the final number of SAH patients and control patients for serum Interleukin (IL)-10 analysis.

**Figure 2:**
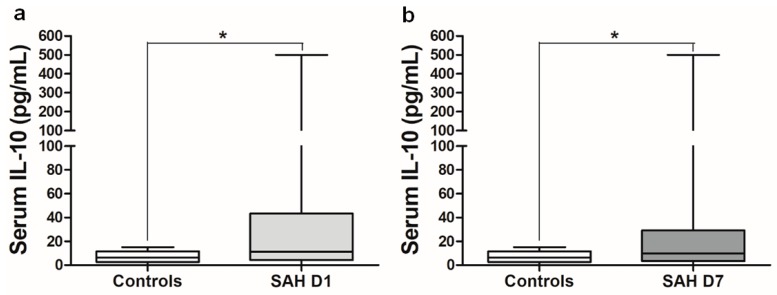
Serum IL-10 levels in control and SAH patients. (**a**) Serum IL-10 levels in control (*n* = 24) and SAH patients (*n* = 76) on day 1. (**b**) Serum IL-10 levels in control (*n* = 24) and SAH patients (*n* = 76) on day 7. Mann–Whitney U test; *p* = 0.011 on day 1, *p* = 0.037 on day 7. (SAH D1 = aneurysmal subarachnoid hemorrhage on day 1, SAH D7 = post aneurysmal subarachnoid hemorrhage day 7) (* *p* < 0.05).

The baseline characteristics of the SAH patients are presented in Table 1. The flow diagram represents the final number of SAH patients recruited into the study (Figure 1). The median for both Hunt and Hess (H&H) and Fischer scores was 3; median Glasgow Outcome Scale (GOS) score at discharge was 4, and median modified Rankin Scale (mRS) score was 3. Serum IL-10 levels were significantly higher in SAH patients on post-SAH day 1 and day 7 compared to the control patients (Figure 2). In the SAH patients, serum IL-10 levels on day 1 were similar to those on day 7 (Figure 2).

Dichotomization of the data based on gender, aneurysm location or treatment modality (data not shown) revealed no significant difference in serum IL-10 levels. Serum IL-10 levels were slightly, but nonsignificantly higher in patients older than 55 years when sampled on day 1 post-SAH (Figure S1a). This difference reached significant, higher levels on day 7 post-SAH (Figure S1b). Serum IL-10 levels were unchanged in SAH patients with poor H&H grades (i.e., 3–5) compared to those with good grades (i.e., 1 or 2; data not shown) on day 1 and day 7 post-SAH. Serum IL-10 levels were significantly higher on day 7 in patients who presented with additional IVH compared to those without IVH (Figure S1d). Serum IL-10 levels were significantly higher on day 1 post-SAH in patients with intracerebral bleeding (ICB) only when this complication was accompanied by intraventricular hemorrhage (IVH); ICB alone did not change the serum IL-10 levels (Figure S1e).

### 2.2. Serum IL-10 Levels were Elevated in Patients with post-SAH Complications

**Figure 3:**
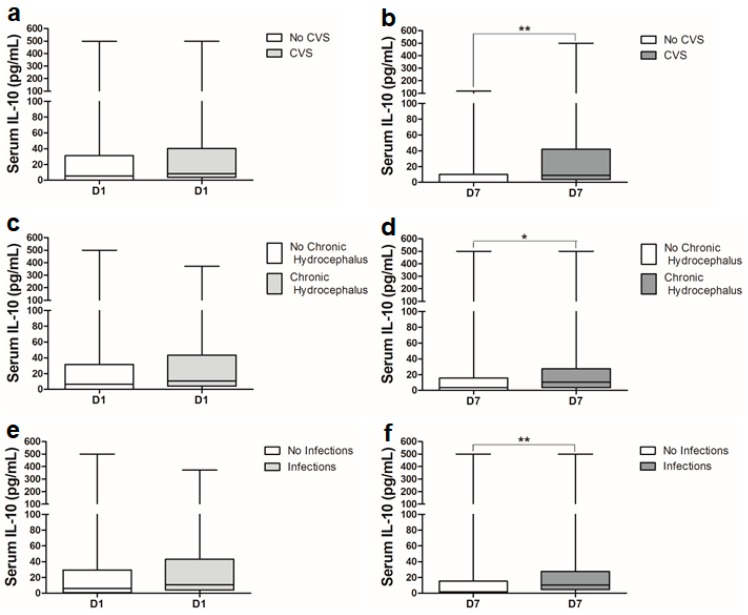
Comparison of serum IL-10 levels between SAH patients with: (**a**) no CVS (*n* = 32) and CVS (*n* = 44) on day 1; (**b**) no CVS (*n* = 32) and CVS (*n* = 44) on day 7; (**c**) no VP-shunt-dependent chronic hydrocephalus (*n* = 53) and VP-shunt-dependent chronic hydrocephalus (*n* = 23) on day 1; (**d**) no VP-shunt-dependent chronic hydrocephalus (*n* = 53) and VP-shunt-dependent chronic hydrocephalus (*n* = 23) on day 7; (**e**) no infections (*n* = 49) and infections (*n* = 27) on day 1; (**f**) no infections (*n* = 49) and infections (*n* = 27) on day 7. Mann–Whitney U test; *p* < 0.05 is significant (* *p* < 0.05; ** *p* < 0.01).

**Figure 4:**
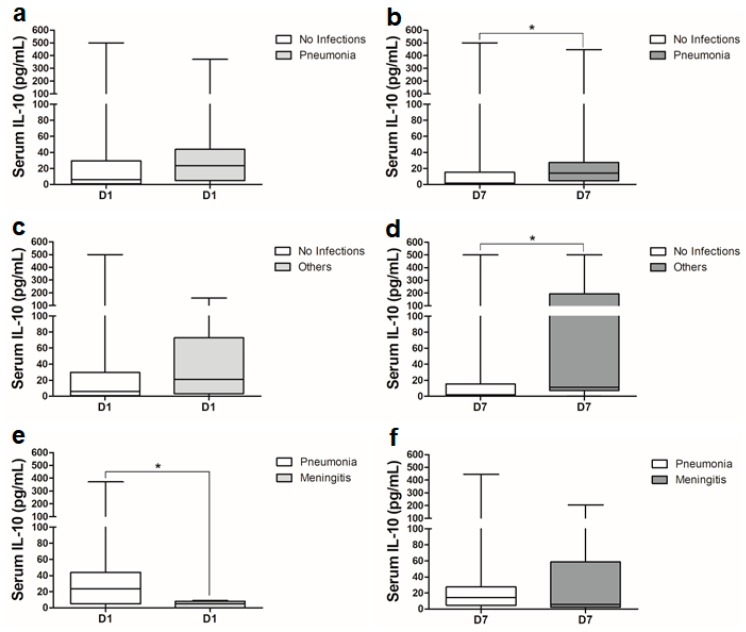
Comparison of serum IL-10 levels between SAH patients with: (**a**) no infections (*n* = 49) and pneumonia (*n* = 15) on day 1; (**b**) no infections (*n* = 49) and pneumonia (*n* = 15) on day 7; (**c**) no infections (*n* = 49) and others (*n* = 6) on day 1; (**d**) no infections (*n* = 49) and others (*n* = 6) on day 7; (**e**) pneumonia (*n* = 15) and meningitis (*n* = 6) on day 1; (**f**) pneumonia (*n* = 15) and meningitis (*n* = 6) on day 7. Mann–Whitney U test; *p* < 0.05 is significant (* *p* < 0.05).

Dichotomization of the data based on SAH-associated complications revealed altered serum IL-10 levels in patients with post-SAH complications (Figure 3 and Figure 4). Patients who experienced convulsive seizures and were treated with anti-epileptic drugs had slightly, but nonsignificantly higher serum IL-10 levels on day 1 and day 7 compared to patients without seizures (data not shown). There was a significant elevation in serum IL-10 levels on day 7 in SAH patients who developed cerebral vasospasm (CVS), nosocomial infections or shunt-dependent chronic hydrocephalus (Figure 3b,d,f). The ROC curve analysis showed a significant area under the curve (AUC = 0.719) at a cutoff value of 2.36 pg/mL associated with 80% sensitivity and 63% specificity for the prediction of CVS on day 7 (Figure S2d). At a cutoff value of 6.07 pg/mL, serum IL-10 levels on day 7 predicted the development of chronic hydrocephalus with 70% sensitivity and 60% specificity (Figure S3a). Serum IL-10 levels were slightly, but nonsignificantly higher in patients with delayed cerebral ischemia (DCI) compared to the patients without DCI. Interestingly, serum IL-10 levels were significantly higher on day 7 in SAH patients who contracted different infections during acute treatment period of SAH (Figure 3f). ROC analysis showed a significant AUC of 0.688 and predicted post-SAH infection with 70% sensitivity and 61% specificity at a cutoff value of 5.44 pg/mL on day 7 (Figure S3b). Further analysis of data based on the type of infection showed that serum IL-10 levels were significantly higher on day 7 in patients who developed pneumonia or other infections (urinary tract infections, or any other infections in combination with pneumonia) (Figure 4b,d) in comparison to the patients who did not develop any kind of infection during the hospital stay. Very interestingly, serum IL-10 levels were already very high on admission in SAH patients who developed later pneumonia compared to the patients who developed local infections (Figure 4e).

### 2.3. Serum IL-10 Levels were Elevated in Patients with Poor Clinical Outcome

**Figure 5:**
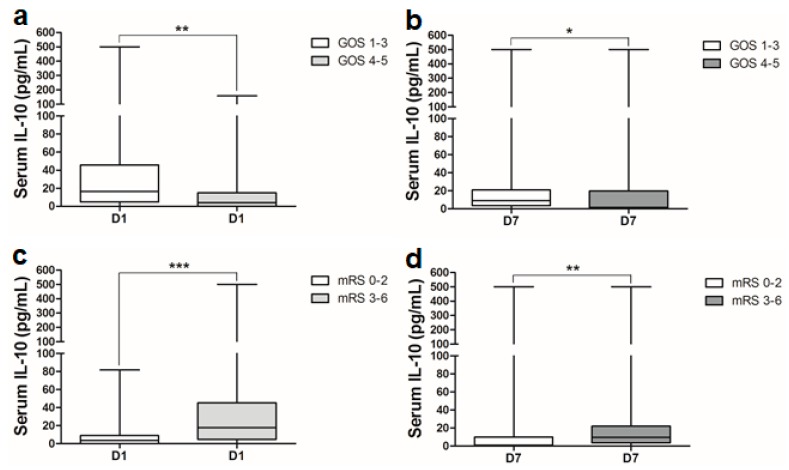
Comparison of serum IL-10 levels between SAH patients with: (**a**) good clinical outcome (GOS 4–5, *n* = 40) and poor outcome (GOS 1–3, *n* = 36); (**b**) good clinical outcome (GOS 4–5, *n* = 40) and poor outcome (GOS 1–3, *n* = 36); (**c**) good clinical outcome (mRS 0–2, *n* = 35) and poor outcome (mRS 3–6, *n* = 41); (**d**) good clinical outcome (mRS 0–2, *n* = 35) and poor outcome (mRS 3–6, *n* = 41). Mann–Whitney U test; *p* < 0.05 is significant (* *p* < 0.05; ** *p* < 0.01; *** *p* <0.001).

Serum IL-10 levels were significantly higher on both day 1 and day 7 post-SAH in patients with poor clinical outcome (GOS 1–3) compared to the patients with good clinical outcome (GOS 4–5) (Figure 5a,b). Clinical outcome was also assessed using another test battery, the modified Rankin Scale (mRS), that also revealed similar results with higher serum IL-10 levels in patients with poor clinical outcome at discharge (mRS 3–6) compared to those with good outcome (mRS 0–2) (Figure 5c,d). ROC analysis for the prediction of poor clinical outcome (assessed with the GOS scores and mRS scores) showed a significant AUC (Figure S3c–f). Clinical outcome on day 1 was predicted with a sensitivity of 72% and specificity of 60% (cutoff value 5.9 pg/mL).

## 3. Discussion 

Our investigations show elevated systemic IL-10 levels after SAH. Among SAH patients who developed any kind of infection, those with CVS or chronic shunt-dependent hydrocephalus had higher serum IL-10 levels compared to those who did not develop these complications. The patients who developed pneumonia later on had already very high serum IL-10 levels on admission, and higher serum IL-10 levels predicted a poor clinical outcome at discharge from the hospital. Our data showing elevated systemic IL-10 levels following SAH are consistent with the existing evidence in literature and explore novel findings on elevated IL-10 and occurrence of nosocomial infections following SAH.

Neuronal injury and the associated neurological outcome is based on a delicate balance between proinflammatory and anti-inflammatory mediators [22]. For example, the proinflammatory cytokine IL-6 can induce an anti-inflammatory response by upregulating IL-10 at the cellular level via regulatory mechanisms, and the correlation between serum IL-6 and IL-10 levels after intracerebral bleeding has been well documented [23,24]. Recently, this correlation between serum IL-6 and IL-10 was also shown under different post-SAH conditions [20]. Accordingly, our study confirmed that IL-6 levels were significantly correlated with IL-10 levels on both days of assessment after SAH (Figure S2a,b). Most of the previous clinical studies have quantified serum IL-10 levels in CSF or extracellular fluid only from SAH patients (sampled by microdialysis) without comparison to the control conditions [17,18,19,20,25]. We thus included control patients in the current study. Compared to control patients, serum IL-10 levels were significantly higher in SAH patients on both days of assessment (Figure 2). This increase in serum IL-10 after SAH is in agreement with results found in the literature [26]. Subgroup analysis after dichotomization showed no impact of patient’s gender, aneurysm location or treatment modality on serum IL-10 levels. This finding is consistent with the results of another previous study [19]. We observed a marked elevation in serum IL-10 levels in SAH patients older than 55 years on post-SAH day 7 (Figures S1a,b). This might reflect an overall IL-10 upregulation in response to a global inflammatory response following SAH, since no such difference exists in comparisons between young and older subjects that are healthy [27,28,29,30]. Moreover, this global inflammatory response might be more severe in elderly patients and might contribute to poor outcome at discharge. It has been shown previously that SAH patients with poor grades (H&H ≥ 4) have significantly elevated peripheral IL-10 levels [20,31,32]. We observed a nonsignificant increase in serum IL-10 levels in poor-grade SAH patients compared to good-grade patients (data not shown). This difference might be a result of differences in H&H grades used between studies, since the earlier study considered higher H&H grades (≥4) to represent clinically severe SAH patients, whereas we classified poor H&H grades above 2 (3–5). Interestingly, patients with intraventricular hemorrhage or a combined IVH and ICB (Figure S1e) had higher IL-10 levels on admission, showing the influence of IVH in the brain, i.e., that it may control systemic IL-10 release through brain–immune-system interactions. The additive effect of ICB on IL-10 levels is consistent with results from earlier studies that also consistently show a significant increase in IL-10 after ICB. Serum IL-10 levels on day 7 were higher in SAH patients who developed CVS (Figure 3b). Similar findings were reported previously [33]. An increase in IL-10 expression has been shown to be associated with a parallel surge of proinflammatory cytokines (IL-1β, TNF-α and IL-6) and increased high-mobility group box-1 (HMGB1) expression. Glycyrrhizic acid supplementation not only relieved vasospasm, but also inhibited the expression of proinflammatory factors and further enhanced IL-10 expression [33]. Our earlier work has also shown a significant elevation of HMGB1 in SAH patients presenting with CVS [7]. HMGB1 signaling via RAGE, specifically involving monocytes/macrophages, is known to enhance brain damage after ischemia [34]. HMGB1 is also known to selectively drive IL-10 release from M2-like macrophages through RAGE signaling [35]. Our data show that IL-10 on day 7 correlates with HMGB1 levels on day 1, suggesting that early elevated HMGB1 may influence IL-10 release (Figure S2c).

Serum IL-10 levels were significantly higher on day 7 in patients with shunt dependent hydrocephalus (Figure 3d). In the context of hemorrhagic stroke and hydrocephalus, plasma IL-10 levels are significantly associated with adverse outcomes like re-bleeding and hematoma expansion [36,37]. Higher intracranial pressure is also known to elevate levels of other anti-inflammatory cytokines [38]. Post-SAH nosocomial infections have been reported in around 30% of SAH patients [4,39]. IL-10 is a well-known master regulator of immunodepression leading to systemic infections. Serum IL-10 levels were significantly higher on day 7 in patients with systemic or local infection (Figure 3f). Further analysis of different types of infections showed that this significant rise in IL-10 on day 7 could be an indication of immunodepression that later increased the probability to develop pneumonia or other infections (UTI, osteomyelitis or concomitant presence of these with pneumonia or meningitis) (Figure 4a–f). Our data are consistent with previous observations [32]. Very interestingly, a significant increase in serum IL-10 levels was observed already on day 1 in patients who developed pneumonia in comparison to patients without infections or who developed only a local infection (Figure 4e).

Prediction of clinical outcome is important for clinical decision-making. Hence, we assessed outcome at discharge using GOS and mRS. Our results showed that serum IL-10 levels on both days of assessment were significantly higher in patients with poor clinical outcome (Figure 5a–d). Comprehensive studies investigating serum IL-10 levels and its association with post-SAH infection, immunodepression and clinical outcome are lacking [21]. To the best of our knowledge, this is the first study to associate serum IL-10 levels with poor clinical outcome at discharge during the period of early brain injury (day 1) and delayed brain injury (day 7). Only a single study showed an association between serum IL-10 and clinical outcome (using good outcome mRS scores ≤ 3), but these samples were only analyzed at a single time point [20]. These novel investigations that report upregulation of systemic IL-10 after SAH are consistent with the results of earlier investigations of ischemic stroke, intracerebral hemorrhage or traumatic brain injury [19,21,38,40,41].

IL-10 represents an important anti-inflammatory cytokine secreted by almost all immune cells [12]. An increase in IL-10 levels is usually associated with the elevation of proinflammatory cytokines (such as TNF-α, IL-1β and IL-6) [12,20,33]. Therefore, the significant elevation of IL-10 after SAH may reflect an upregulated inflammatory response and immunodepression that is triggered by SAH [20,42]. Taken together, high IL-10 serum levels predicted infections and poor outcome after SAH; this urges further investigation as to whether elevated IL-10 leads to immunodepression at the level of cellular capacity to present antigens or if it represents as marker of ongoing inflammatory response to injury. Our study has interesting findings but has some limitations. First, our patient population is heterogeneous with a wide age range, representation of both sexes, and diverse grades of SAH severity (i.e., H&H grades ranging from 1 to 5) potentially creating a low signal-to-noise ratio. Second, the small sample sizes in the subgroup analysis may have lowered the statistical ability to detect significant differences. Moreover, early post-SAH infections before day 7 may also confound IL-10 levels. The multitude of IL-10 producing cells with complex feed-forward and -backward loops and potential to promote adaptive immune response, in addition to potent anti-inflammatory properties, short half-life and limited range of activity, requires translational studies in SAH models focusing on replenishing and protecting endogenous IL-10 levels. Additionally, neutralization of IL-10 in SAH models will advance our understanding of the pleiotropic effects of IL-10 in SAH [43]. Hence, the conclusion should be interpreted carefully. Moreover, validation in larger groups of patients would be required for implications in the clinical setting.

## 4. Materials and Methods

### 4.1. Patient Population

During 2012–2016, we prospectively enrolled 76 SAH patients with Hunt and Hess (H&H) grades of 1–5 and Fischer scores of 1–4. The patients presenting within 24 h of SAH were considered for sampling. Patients were excluded if they presented the following: ischemic stroke, traumatic brain injury, onset of symptoms beyond 24 h, SAH due to arteriovenous malformations or vasculitis, pregnancy, signs of eminent death, under 18 years old, or they did not provide consent. The choice of aneurysm treatment (either neurosurgical clipping or endovascular coiling) was based on an interdisciplinary decision. Computed tomography (CT)-angiography, CT-perfusion, or digital subtraction angiography was performed whenever CVS was suspected. Patients were considered for CVS treatment if their vascular diameter decreased by more than 50% and if they had a prolonged mean transit time (MTT) above 5 s or prolonged more than 2 s in comparison to the contralateral side. Delayed ischemic neurological deficits (DIND) displayed by alterations in consciousness, hemiparesis or aphasia were considered as neurological worsening. Cerebral ischemia (CI) denoted cerebral infarction as assessed by cranial CT, and delayed cerebral ischemia (DCI) represents cerebral ischemia that cannot be attributed to neurosurgical or endovascular aneurysm repair. Glasgow Outcome Scale (GOS) and modified Rankin Scale (mRS) were used as clinical outcome measures and were prospectively recorded at discharge. The study was conducted according to the guidelines of the Declaration of Helsinki and approved by the local ethical committee of the Faculty of Medicine (University of Bonn, Germany; Reference Number: LfD 138/2011 on 02.08.2011). Informed consent was signed by the patients/guardians and obtained from the treating neurosurgeon.

### 4.2. Sample Collection and Analysis

Peripheral venous blood was withdrawn into serum gel tubes (Monovette, Sarstedt, Nuembrecht, Germany) on day 1 and day 7 post-SAH and withdrawn once from the control patients. Blood was centrifuged at 3000 rpm in a benchtop centrifuge (Sigma, Osterode am Harz, Germany) for 10 min. Serum was isolated and immediately frozen at −80 °C until analysis. Serum IL-10 levels were determined by using precoated ELISA kits (BD OptEIA^TM^, CA, USA) following the manufacturer’s instructions.

### 4.3. Statistical Analysis

Serum IL-10 levels were represented with box and whisker plots. Serum IL-10 levels between the two groups (control and SAH patients) were compared using a Mann–Whitney U test. Within the SAH patients, levels between day 1 and day 7 post-SAH were compared using a Wilcoxon signed-rank sum test. For subgroup analysis of different characteristics of the SAH patients (age, gender, H&H and Fischer scores, aneurysm treatment modality, aneurysm location, intraventricular bleeding (IVH) and intracerebral bleeding (ICB)), data were dichotomized into two groups (e.g., age < 55 years old vs. ≥ 55 years old; mild vs. severe SAH) and analyzed with a Mann–Whitney U test. The same was done for post-SAH complications (CVS, DIND, chronic hydrocephalus, seizures, infections, CI) and clinical outcomes (GOS, mRS). The data were analyzed using GraphPad Prism 5.00 (GraphPad Software, San Diego, CA, USA). Receiver operating characteristic (ROC) curve analysis was performed using SPSS (IBM SPSS version 24 for Windows, IBM Corp., Armonk, NY, USA).

## 5. Conclusions

Aneurysmal subarachnoid hemorrhage induced systemic IL-10 release. Serum IL-10 levels at day 7 were significantly higher in SAH patients who developed any kind of infection, CVS or shunt-dependent hydrocephalus. Serum IL-10 levels were already very high on admission in patients who developed later pneumonia. Patients with poor clinical outcome (mRS 4–6 or GOS 1–3) at discharge revealed significantly higher IL-10 levels. Elevated serum IL-10 levels may indicate signs of immunodepression and infection contributing to poor outcome after SAH.

## Figures and Tables

**Table 1:** Characteristics of aneurysmal subarachnoid hemorrhage (SAH) patients.

**Number of SAH Patients**	**76**
**Age** (years) (mean ± SD)	**59.14** (**±11.93**)
**Females** (**%**)	**60.53%**
**Treatment modality**	
Neurosurgical clipping (%)	48.7%
Endovascular coiling (%)	51.3%
**Intraventricular hemorrhage (IVH)** (%)	**13.2%**
**Intracerebral bleeding (ICB)** (**%**)	**18.4%**
**ICB and IVH** (**%**)	**14.5%**
**Hunt and Hess Grade** (**Median**)	**3**
1 (%)	7.9%
2 (%)	31.6%
3 (%)	28.9%
4 (%)	15.8%
5 (%)	15.8%
**Fischer Grade** (**Median**)	**3**
1 (%)	1.3%
2 (%)	2.6%
3 (%)	82.9%
4 (%)	13.2%
**Cerebral vasospasm (CVS)** (**%**)	**57.9%**
**Cerebral Ischemia (CI)** (**%**)	**43.4%**
**Delayed Cerebral Ischemia (DCI)** (**%**)	**21.1%**
**Seizures** (**%**)	**28.9%**
**VP-Shunt-dependent hydrocephalus** (**%**)	**30.3%**
**Infections** (**%**)	**35.5%**
Pneumonia (%)	19.7%
Meningitis (%)	7.9%
Others (Pneumonia, Meningitis in combination with UTI, or Osteomyelitis, wound infection) (%)	7.9%
**DIND** (**%**)	**34.2%**
**Aneurysm location**	
Anterior circulation (%)	85.5%
Posterior circulation (%)	14.5%
**Glasgow Outcome Scale (GOS)** (**median**)	**4**
1 (%)	7.9%
2 (%)	11.8%
3 (%)	27.6%
4 (%)	7.9%
5 (%)	44.7%
**Modified Rankin Scale (mRS)** (**median**)	**3**
0 (%)	2.6%
1 (%)	34.2%
2 (%)	9.2%
3 (%)	9.2%
4 (%)	19.7%
5 (%)	17.1%
6 (%)	7.9%

VP-Shunt-dependent hydrocephalus: Ventriculoperitoneal-Shunt-dependent hydrocephalus; DIND: Delayed Ischemic Neurological Deficits.

## Supplementary Materials

Supplementary Materials can be found at https://www.mdpi.com/1422-0067/21/5/1569/s1.

## Abbreviations

SAH	Aneurysmal Subarachnoid Hemorrhage
mRS	Modified Rankin Scale
GOS	Glasgow Outcome Scale
RANTES	Regulated on activation, normal T cell expressed and secreted
CVS	Cerebrovascular spasm
DIND	Delayed ischemic neurological deficits
CI	Cerebral ischemia (infarction)
DCI	Delayed Cerebral Ischemia
ICB	Intracerebral bleeding
IVH	Intraventricular hemorrhage
TNF	Tumor necrosis factor
PAF	Platelet activating factor
LPS	Lipopolysaccharide
DCs	Dendritic cells
Tregs	CD4+ T regulatory cells
PBMCs	Peripheral blood mononuclear cells
MTT	Mean transit time
CT	Computerized tomography
