# Supplementary material for: Elevated Systemic IL-10 Levels Indicate Immunodepression Leading to Nosocomial Infections after Aneurysmal Subarachnoid Hemorrhage (SAH) in Patients
Source: Int J Mol Sci. 2020 Feb 25;21(5):1569. doi: 10.3390/ijms21051569 (PMC7084744; doi:10.3390/ijms21051569)
Supplement: Supplementary file 1 [file ijms-21-01569-s001.pdf]

Supplementary material

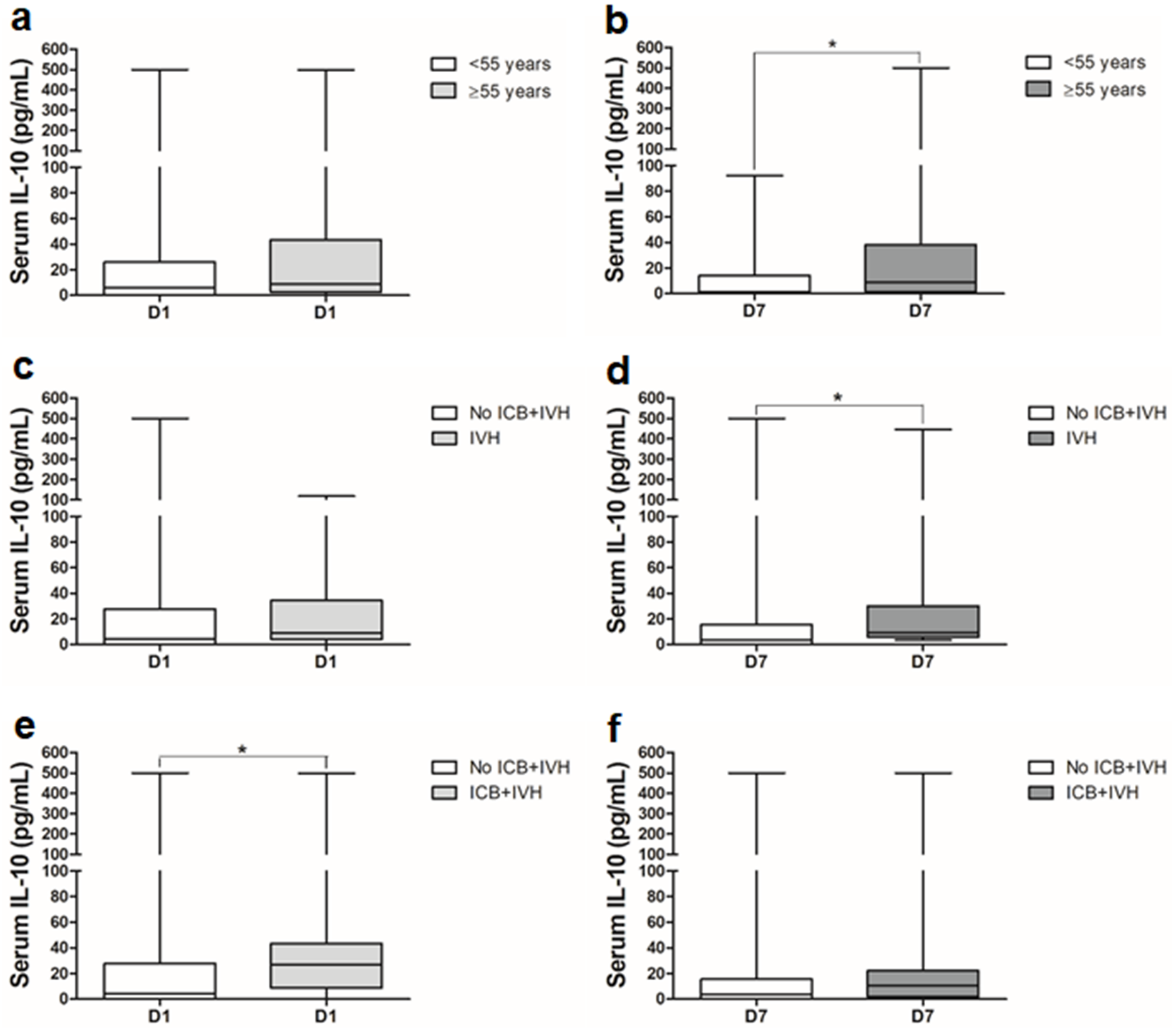

Figure S1. Comparison of serum IL-10 levels between SAH patients with: (**a**)**.** age <55 years (n = 29) and ≥ 55 years (n = 47) on day 1; (**b**)**.** age <55 years (n = 29) and ≥ 55 years (n = 47) on day 7; **(c).** no intracerebral bleeding and intraventricular hemorrhage (No ICB+IVH; n = 41), and intraventricular hemorrhage (IVH; n = 10) on day 1; **(d).** no intracerebral bleeding and intraventricular hemorrhage (No ICB+IVH; n = 41), and intraventricular hemorrhage (IVH; n = 10) on day 7 **(e).** no ICB and IVH (n = 41) and both IVH and ICB (n = 11) on day 1; **(f).** no ICB and IVH (n = 41) and both IVH and ICB (n = 11) on day 7. Mann Whitney U test; p value < 0.05 is significant *(* p < 0.05).*

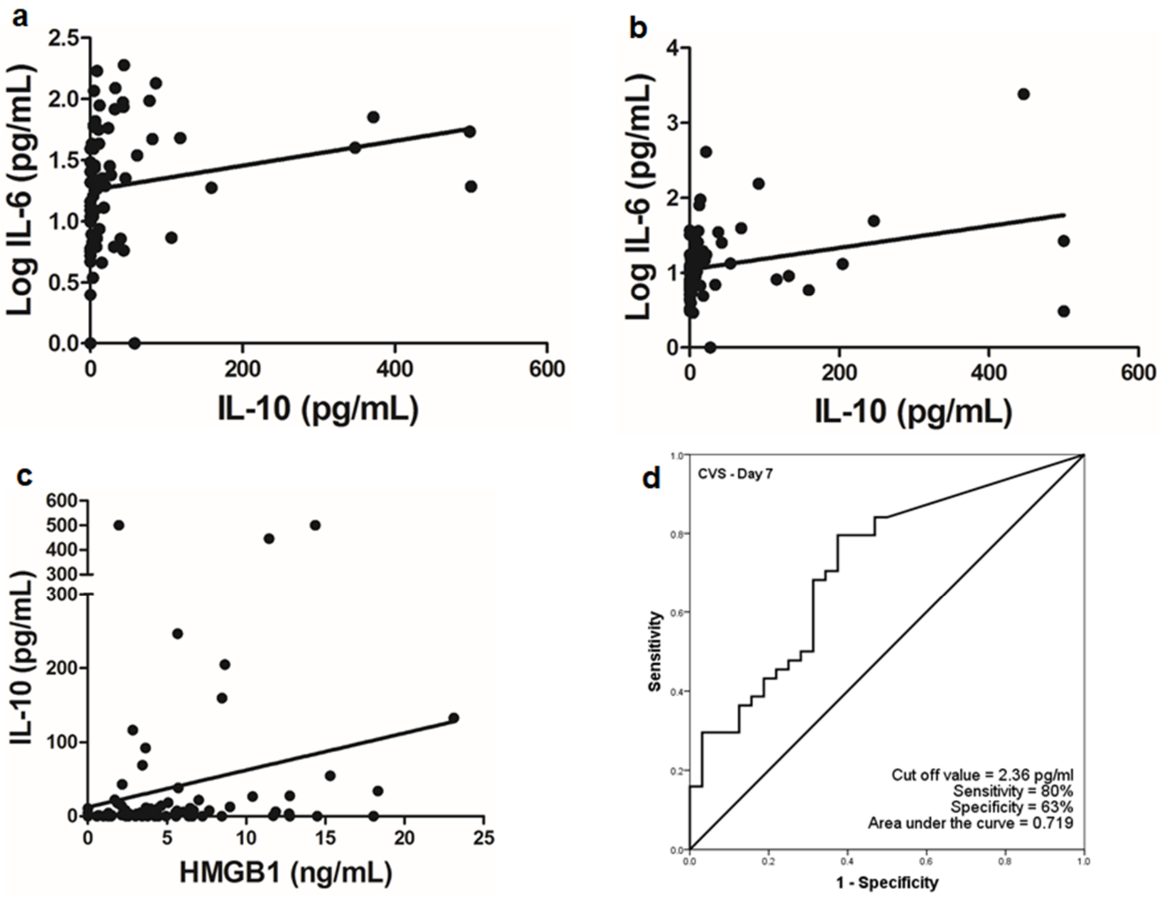

Figure S2: Correlation between IL-10 and IL-6 serum levels on: **(a).** day 1 post-SAH (n = 74, Spearman's rho = 0.390, p = 0.001); **(b).** day 7 post-SAH (n = 75, Spearman's rho = 0.418, p = <0.000); **(c).** Correlation between High mobility group box-1 (HMGB1) on day 1 and serum IL-10 levels on day 7 (n = 72, Spearman's rho = 0.234, p = 0.047). **(d).** ROC curve for the prediction of CVS on day 7 post-SAH.

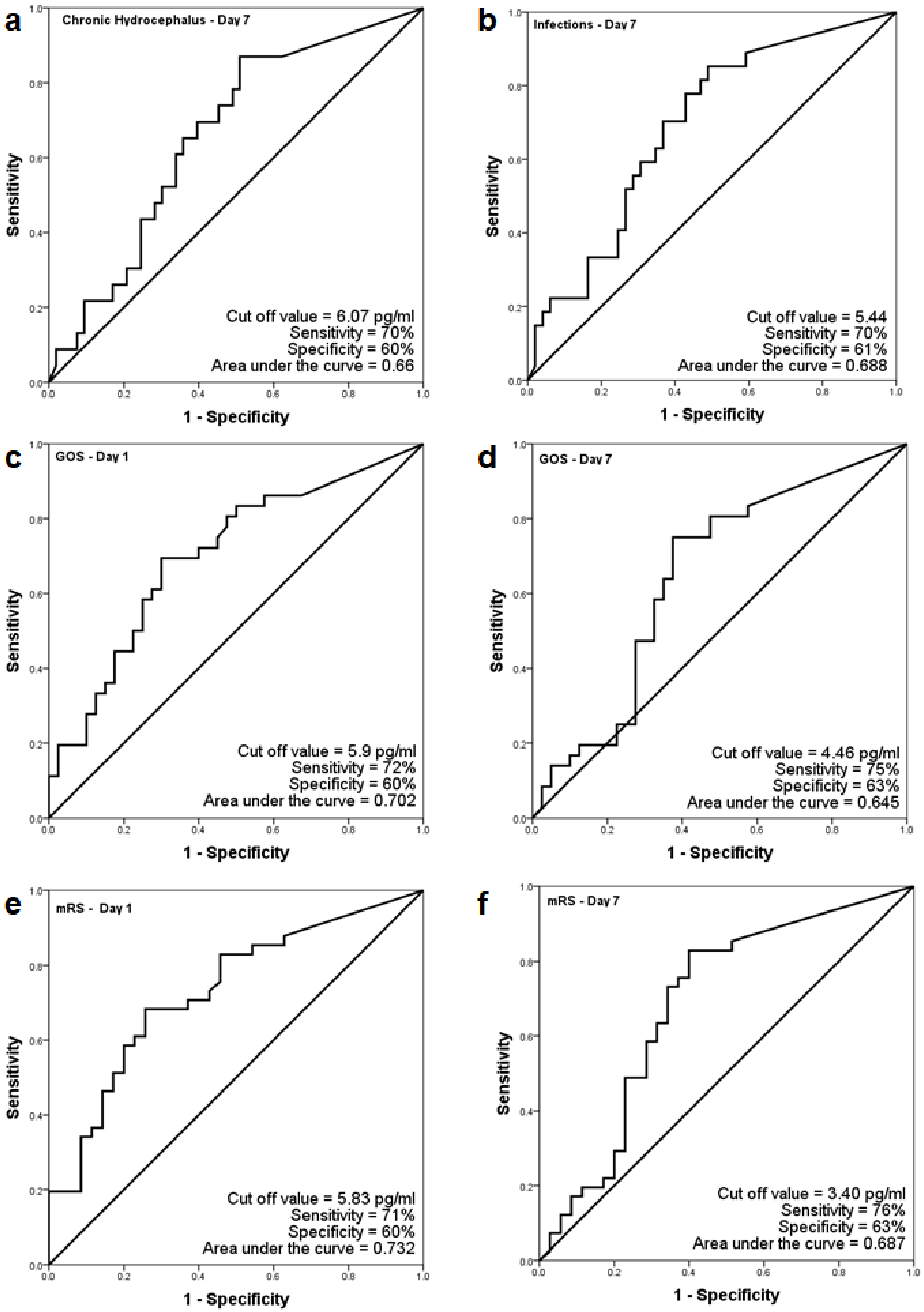

Figure S3: ROC curve for the prediction of: (**a**). chronic hydrocephalus on day 7 post-SAH; (**b**). infections on day 7 post-SAH; **(c).** poor clinical outcome (assessed by GOS) on day 1 post-SAH; (**d**). poor clinical outcome (assessed by

GOS) on day 7 post-SAH; (**e**)**.** poor clinical outcome (assessed by mRS) on day 1 post-SAH. (**f**)**.** poor clinical outcome (assessed by mRS) on day 7 post-SAH
